# Targeting Tumor-Stromal Interactions in Pancreatic Cancer: Impact of Collagens and Mechanical Traits

**DOI:** 10.3389/fcell.2021.787485

**Published:** 2021-11-25

**Authors:** Parniyan Maneshi, James Mason, Mitesh Dongre, Daniel Öhlund

**Affiliations:** ^1^ Department of Radiation Sciences, Umeå University, Umeå, Sweden; ^2^ Wallenberg Centre for Molecular Medicine, Umeå University, Umeå, Sweden

**Keywords:** pancreatic cancer, PDAC—pancreatic ductal adenocarcinoma, stroma, collagen, mechanical traits, extracellular matrix

## Abstract

Pancreatic ductal adenocarcinoma (PDAC) has one of the worst outcomes among cancers with a 5-years survival rate of below 10%. This is a result of late diagnosis and the lack of effective treatments. The tumor is characterized by a highly fibrotic stroma containing distinct cellular components, embedded within an extracellular matrix (ECM). This ECM-abundant tumor microenvironment (TME) in PDAC plays a pivotal role in tumor progression and resistance to treatment. Cancer-associated fibroblasts (CAFs), being a dominant cell type of the stroma, are in fact functionally heterogeneous populations of cells within the TME. Certain subtypes of CAFs are the main producer of the ECM components of the stroma, with the most abundant one being the collagen family of proteins. Collagens are large macromolecules that upon deposition into the ECM form supramolecular fibrillar structures which provide a mechanical framework to the TME. They not only bring structure to the tissue by being the main structural proteins but also contain binding domains that interact with surface receptors on the cancer cells. These interactions can induce various responses in the cancer cells and activate signaling pathways leading to epithelial-to-mesenchymal transition (EMT) and ultimately metastasis. In addition, collagens are one of the main contributors to building up mechanical forces in the tumor. These forces influence the signaling pathways that are involved in cell motility and tumor progression and affect tumor microstructure and tissue stiffness by exerting solid stress and interstitial fluid pressure on the cells. Taken together, the TME is subjected to various types of mechanical forces and interactions that affect tumor progression, metastasis, and drug response. In this review article, we aim to summarize and contextualize the recent knowledge of components of the PDAC stroma, especially the role of different collagens and mechanical traits on tumor progression. We furthermore discuss different experimental models available for studying tumor-stromal interactions and finally discuss potential therapeutic targets within the stroma.

## 1 Introduction

Pancreatic cancer is one of the most fatal malignancies with a 5-years survival rate below 10% (Europe 2018) (Siegel et al., 2021). The poor survival rate in pancreatic cancer is mainly due to its asymptomatic progression in early stages, resulting in late diagnosis, and the lack of effective treatment regimens against the advanced stages of the disease. More than 90% of all pancreatic cancer patients present exocrine tumors where an overwhelming majority of these tumors are pancreatic ductal adenocarcinoma (PDAC) which can originate from either the epithelial cells lining pancreatic ducts or the acinar cells of the pancreas (Flowers et al., 2021) (Grant et al., 2016).

**FIGURE 1:**
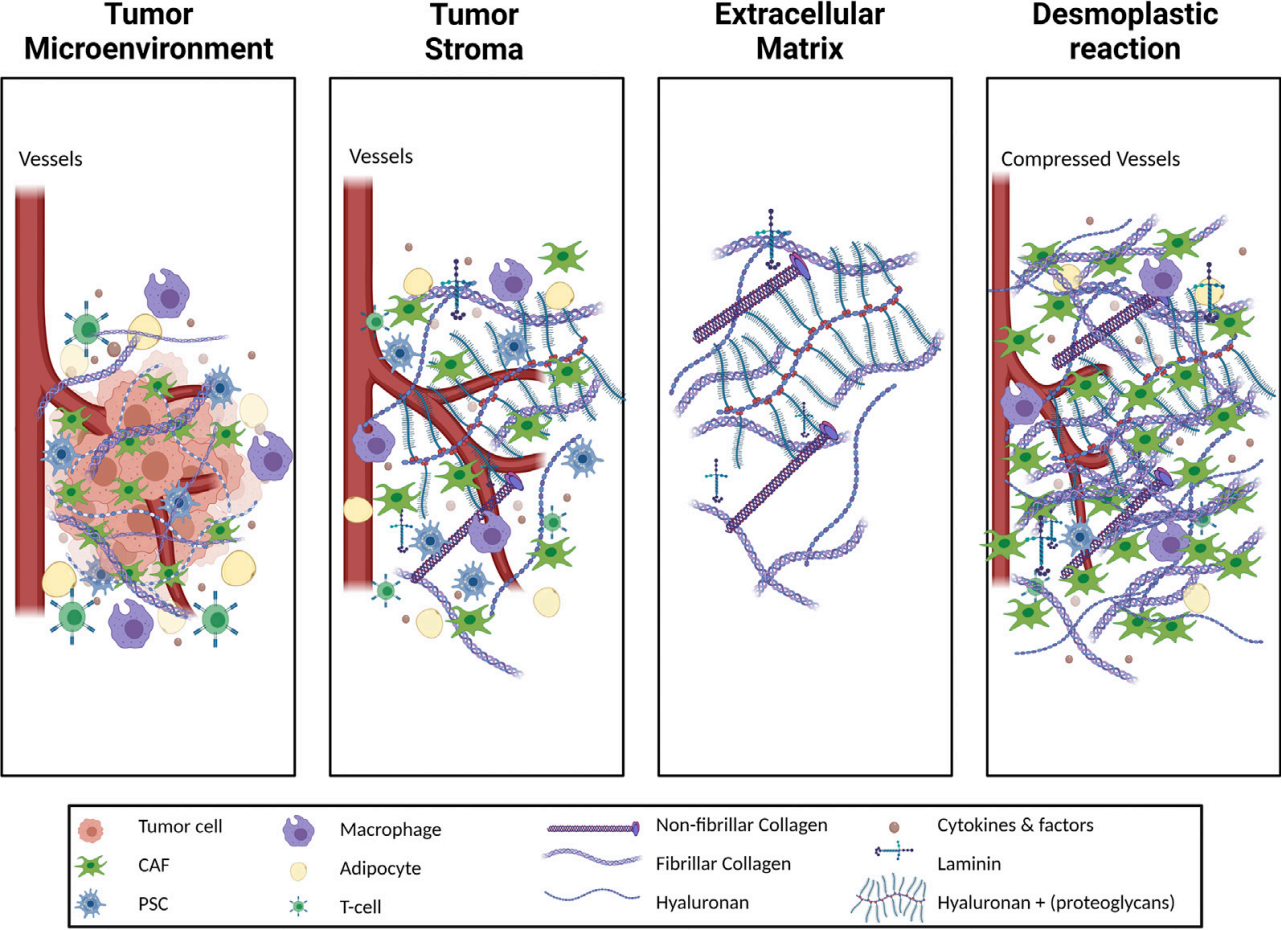
Schematic illustration of different terms used to describe the tumor stroma. The tumor microenvironment (TME) of pancreatic adenocarcinoma (PDAC) includes the tumor cells in addition to the stroma. The stroma is a complex milieu of non-tumor cells, cytokines, growth factors and the proteins of extracellular matrix (ECM). The ECM consists of the non-cellular structural arrangement of the tissue and is highly proteinaceous. Cellular components of PDAC stroma include immune cells such as T-cells and macrophages, as well as stromal cells such as adipocytes and pancreatic stellate cells (PSCs) which are a source of cancer associated fibroblasts (CAFs). CAFs and, to a lesser extent, tumor cells alter the ECM composition by means of increased collagen, both fibrillar and non-fibrillar, deposition as well as laminins, hyaluronan and other proteoglycans. The pathophysiological increase of ECM protein content in the TME results in a desmoplastic reaction, generating denser, stiffer tissue than in the healthy pancreatic setting. Consequences of the desmoplastic reaction are a constrained blood vessels, infiltration around the tissue, and a corresponding reduction of fluid perfusion into the tissue, which in turn cultivates a hypoxic TME.

One of the distinctive characteristics of PDAC, as compared to other solid tumors, is its highly fibrotic stroma which can contribute up to 80% of the tumor volume (Erkan et al., 2012). PDAC stroma primarily consists of extracellular matrix (ECM) and cancer-associated fibroblasts (CAFs) (Figure 1) (Hosein et al., 2020). ECM, which is the three-dimensional (3D) structural non-cellular portion of the tumor, is synthesized and secreted by the cells in the tumor microenvironment (TME) to provide mechanical and biochemical support to the tumor (Frantz et al., 2010). High levels of ECM production in reaction to a pathological state such as a wound, or tumor such as in PDAC, is known as a desmoplastic reaction or desmoplasia. Desmoplasia is further characterized by continuous ECM remodeling which is the constant degradation and deposition of ECM molecules, such as collagens, into the TME. A high amount of collagen deposition in the TME increases tumor density, thus altering its mechanical traits compared to normal pancreatic tissue. These alterations increase solid stress, interstitial fluid pressure, and stiffness in the tumor and collectively bring about changes in the tissue microarchitecture (Nia et al., 2020). These deviations in the mechanical traits of the tumor affect the tumor vasculature resulting in hypoxia and play a significant role in disease progression, invasiveness, metastasis, and treatment resistance (Jain et al., 2014).

In a disease with late-onset symptoms such as PDAC, studies of the precursor lesions become crucial to enable early detection and for improving patient survival. Precursor lesions of PDAC develop as a result of the accumulation of genetic mutations in epithelial cells and include pancreatic intraepithelial neoplasia (PanIN) and intraductal papillary mucinous neoplasm (IPMN). PDAC is presumed to develop primarily from PanIN lesions (Hruban et al., 2004) which share several genetic mutations with PDAC such as a gain of function mutation in the KRAS gene which is found in up to 95% of all PDAC cases (Hruban et al., 2000) and is also found in around 55% of early PanIN lesions and more than 80% of late PanIN (Yonezawa et al., 2008). As with PDAC, desmoplasia is observed in PanIN lesions (Pandol et al., 2012).

The shared characteristic of stromal aberrations in PDAC and in benign ailments which may then develop into PDAC indicates the possibility that stromal aberrations may support or promote tumor initiation and future malignancy. Since desmoplasia is one of the major stromal aberrations common to several precursor lesions as well as PDAC, it is prudent to study the formation of the desmoplastic stroma and its interaction with pancreatic cells in order to understand the role of stroma and changes thereof in the early stages of disease progression. Moreover, it can be surmised that comparing the composition of pancreatic stroma at different stages of the disease might help in identifying unique molecular indicators for the onset of the disease and its progression.

One stromal component that can be studied in detail between disease stages is the matrisome (Tian et al., 2019). The matrisome consists of the full repertoire of ECM proteins, including the core ECM proteins such as collagens, glycoproteins, and proteoglycans, as well as ECM-associated proteins, like ECM regulators, ECM-affiliated proteins, and other secreted metabolites (Naba et al., 2012). It has been shown that collagens are the most prominent group of proteins in the stroma during PDAC development and chronic pancreatitis, accounting for more than 90% of the ECM proteins at all stages (Tian et al., 2019). Collagens are primarily deposited by CAFs into the ECM and provide a hospitable microenvironment for the tumor cells (Bachem et al., 2005) (Olivares et al., 2017). Collagens also play an important role in altering the mechanical traits of tumor tissue (Riegler et al., 2018).

Finding new biomarkers for early PDAC diagnosis in high-risk individuals is under intense exploration. It is suggested that PDAC can take almost two decades to develop from initial mutation to metastatic disease, indicating a significant window of opportunity for early diagnosis (Yachida et al., 2010). Despite this, no PDAC-specific biomarker with high sensitivity for the early disease has yet reached the clinic. One reason for this can be that initial cancer clones stay dormant for a long period of time, and the exponential increase of cancer cells is a late event in disease progression (Hruban et al., 2000) (Notta et al., 2016). However, the remodeling of the stroma starts early and is pronounced already in preinvasive precursor lesions (Erkan et al., 2012) which suggests that stromal fragments in the circulation might be easier to detect than biomarkers released from cancer cells themselves, which is an idea that has shown promising results in initial studies. For example, a fragment of collagen type XVIII, endostatin, has been found in the blood of PDAC patients at higher levels than normal and these levels return to normal after surgery (Öhlund et al., 2008). Another study has shown elevated levels of collagen type IV in the peripheral circulation of PDAC patients and that persisting high levels of collagen type IV after curative surgery is correlated with poor survival and a quick relapse in patients (Öhlund et al., 2009). Moreover, circulating levels of matrix metalloproteinase (MMP)-degraded fragments of collagens type I, III, and IV and a pro-peptide of collagen type III have been used as biomarkers for PDAC (Willumsen et al., 2019). A recent report has also shown the applicability of combined analysis of stroma-derived markers, including collagen type IV and endostatin with conventional tumor markers such as CA 19-9, TPS, CEA, and Ca 125, in detecting PDAC (Franklin et al., 2015).

Taken together, the desmoplastic stroma of PDAC is found early in the development of the disease and plays important and complex pathophysiological roles throughout disease progression. Here, we aim to review recent literature on components of the PDAC stroma, with a focus on the role of different collagens and mechanical traits on tumor progression. Moreover, we discuss different experimental models available for studying tumor-stromal interactions and finally discuss potential therapeutic targets within the stroma.

## 2 The Desmoplastic Reaction in Pancreatic Ductal Adenocarcinoma

The desmoplastic reaction is the formation of a fibrotic, stiff, and collagen-rich connective tissue with high solid stress around the cancer cells (Figure 1). It also creates a special microenvironment that facilitates tumor growth and metastasis while acting as a physical barrier to drug penetration resulting in chemo-resistance (Schober et al., 2014). The complexity and abundance of the ECM increases with an increasing level of desmoplasia as PDAC progresses. (Tian et al., 2019).

**FIGURE 2:**
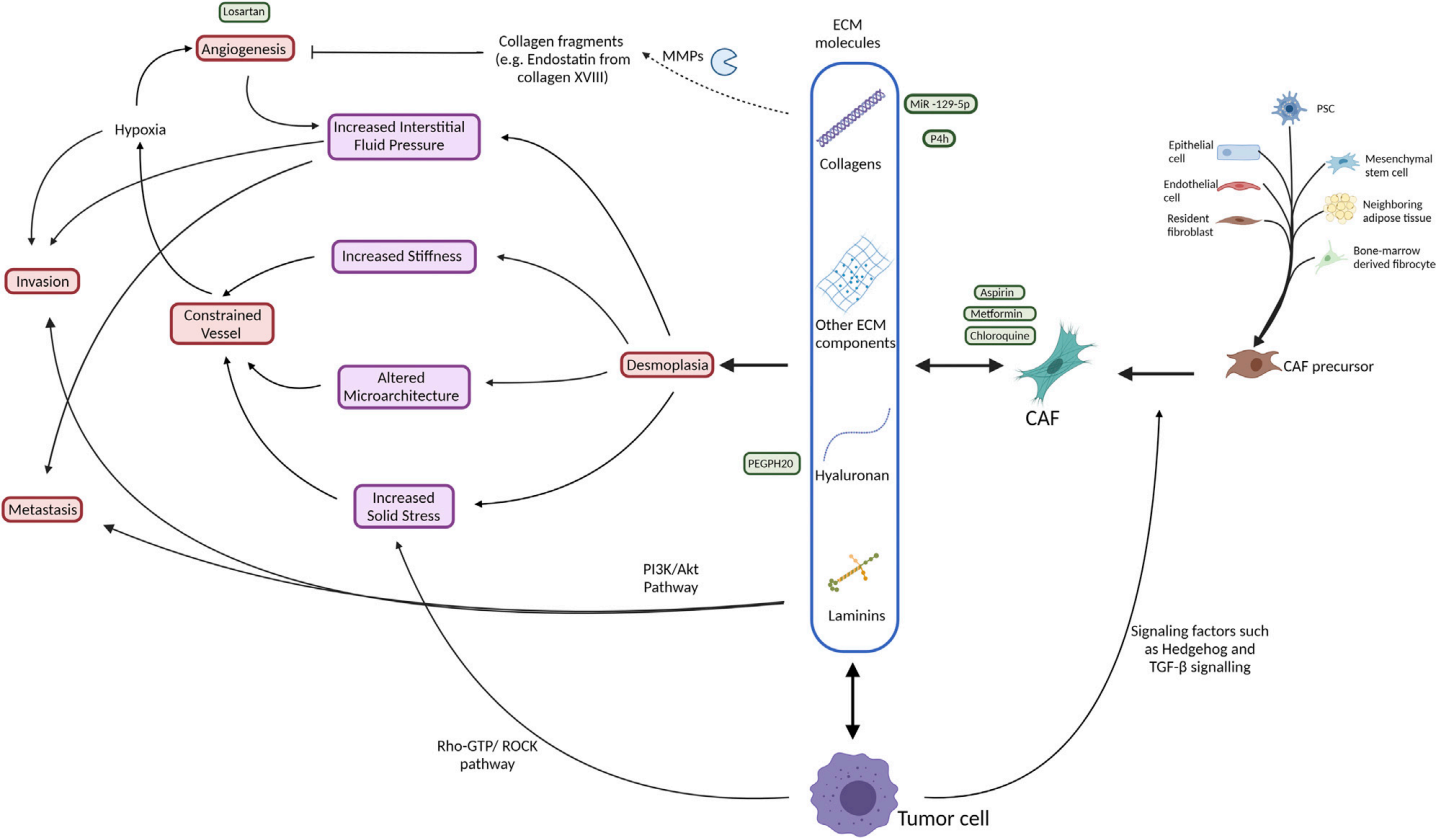
Schematic illustration of the interactive network of the tumor stroma with respect to ECM production (blue box) and its effects on biomechanical (purple) traits and induction of physiological processes (red) within the tissue. Additionally, indicated are various approaches to therapeutic intervention (green). Broadly, both tumor cells and CAFs, which have several cell types of origin, produce ECM proteins that contribute to desmoplasia, consequently increasing tissue stiffness, pressure and architecture in a manner that constrains vessels, leading to a hypoxic environment. This hypoxic environment promotes angiogenesis and cell invasion. The increased angiogenesis results in an increased interstitial fluid pressure that acts to promote invasion and metastasis of the tumor cells. The tumor cells themselves also contribute directly by means of their proliferation to increase the solid stress of the tissue that also supports a feedback loop by constraining vessel access to the tissue. Attempted therapeutic interventions include reduction of CAF formation, degradation of ECM collagens and hyaluronan, MMP inhibition and anti-angiongenic treatments. Akt, protein kinase B; CAF, cancer associated fibroblast; COL, collagen; ECM, extracellular matrix; GTP, guanosine triphosphate; HA, hyaluronan; LAMB3, laminin subunit beta-3; MMP, matrix metalloproteinase; PDAC, pancreatic ductal adenocarcinoma; PEGPH20, PEGylated human recombinant PH20 hyaluronidase; PI3K, phosphatidylinositol 3-kinase; PSC, pancreatic stellate cell; ROCK, Rho associated protein kinase; TGF-β, transforming growth factor β; TME, tumor microenvironment.

The major producers of desmoplasia are fibroblasts. Fibroblasts are the most abundant cell type within the connective tissue with an important role in homeostasis and wound healing (Öhlund et al., 2014). It is hard to define fibroblasts due to their lack of specific markers which are not shared with other lineages, however, they are often identified by their location, morphology and lack of epithelial, endothelial, or leucocyte specific markers (Sahai et al., 2020). They proliferate and differentiate into myofibroblasts in response to tissue injury or cancer, which resembles a “chronic wound” in many ways (Dvorak 1986). This process is marked by increased expression of alpha-smooth muscle actin (α-SMA), a contractile stress fiber protein (Tomasek et al., 2002). CAFs generally refer to all the non-cancerous fibroblastic cells embedded in the ECM of tumor tissue (Öhlund et al., 2014). They are derived from different sources (Figure 2) such as resident fibroblasts (Kojima et al., 2010), bone marrow-derived mesenchymal stem cells (Quante et al., 2011) (Mishra et al., 2008), neighboring adipose tissue (Kidd et al., 2012), or arise from the transdifferentiation of other cell types such as pancreatic stellate cells (PSCs) (Bachem et al., 1998), bone marrow-derived fibrocytes (Reilkoff et al., 2011), epithelial cells (Iwano et al., 2002), and endothelial cells (Zeisberg et al., 2007). Stellate cells are a form of retinoic acid (a metabolite of vitamin A) and lipid-containing cells in their quiescent state that reside in the pancreas among other organs and share some characteristics with fibroblasts (Kordes et al., 2009). Upon activation in response to tissue damage signals or in tumor conditions, PSCs lose their reservoir of retinoic acid and promote desmoplasia by differentiating into α-SMA expressing CAFs that secrete large amounts of collagens into the TME (Kocher et al., 2020) (Bachem et al., 1998). Indeed, the level of PSC activation in the stroma is strongly correlated with collagen deposition (Apte et al., 2004) (Bachem et al., 1998).

Desmoplastic areas within the TME also show high levels of collagenase membrane-type 1 MMP (MT1-MMP), a major ECM-degrading enzyme (Shields et al., 2012). This indicates that collagens are not only produced but also degraded in the tumors, which is reflected by the fact that fragments of collagens can be detected in the blood of PDAC patients (Öhlund et al., 2008) (Willumsen et al., 2019). Besides MMPs, collagens are also degraded by other ECM-modifying enzymes produced by CAFs, such as lysyl oxidases (LOX) and LOX-like proteins which catalyze crosslinking between deposited collagen fibers in the tumors, thereby increasing complexity in the 3D framework of the matrix (Myllyharju and Kivirikko, 2004).

The significance of desmoplasia in influencing cancer cell activity is well accepted, but the evidence on whether these factors have a suppressive or supporting role in PDAC is multifaceted (Ligorio et al., 2019). Incubation of PSCs with conditioned media from cancer cells leads to a significant increase in their proliferation (Apte et al., 2004). Similarly, CAF conditioned media has also led to a significant increase in invasive potency of the already invasive PDAC cell lines *in vitro* (Ligorio et al., 2019), indicating an important paracrine crosstalk between cancer cells and the surrounding CAFs. In addition, using a mouse orthotopic xenograft model, a significant increase in the metastatic burden was observed with *in vivo* transplantation of PDAC cell lines together with CAFs compared to PDAC cell lines alone (Ligorio et al., 2019).

Even though most studies on tumor stroma have demonstrated the pro-tumorigenic effects of desmoplasia, newfound evidence has revealed tumor-suppressive features of the stroma. The controversial effects of the stroma on PDAC progression and pathogenesis can be linked to the phenotypic variation of different subtypes of CAFs. We have previously identified two phenotypically distinct, mutually exclusive, and spatially separated CAF subtypes, myofibroblastic CAFs (myCAFs) and inflammatory CAFs (iCAFs) (Öhlund et al., 2017). It is hypothesized that some CAF subtypes may be able to restrain cancer progression while others facilitate it. *In vivo* studies in a mouse model of PDAC have shown that depletion of the α-SMA positive CAFs in PanIN or PDAC, while reducing the desmoplasia, resulted in increased hypoxia, invasiveness, epithelial-mesenchymal transition (EMT), and reduced survival (Özdemir et al., 2014). Moreover, deletion of the *Col1a1* gene, which encodes for the alpha 1 subunit of collagen type I, in α-SMA positive CAFs led to a significant decrease in total stromal collagen type I deposition which results in acceleration of disease and reduced overall survival due to the suppression of CD8^+^ T cells (Chen et al., 2021). In addition, inhibition of a G-protein coupled receptor, smoothened (SMO), in the hedgehog signaling cascade in PDAC tumors led to reduced desmoplasia, decreased collagen type I content, and a transient increase in vascularization that improved the delivery of chemotherapy and immunotherapy to the tumors resulting in longer median survival in genetically engineered animal models of PDAC (Olive et al., 2009) (Wang et al., 2019). On the other hand, despite the ability of Sonic hedgehog (SHH) to drive the formation of a CAF-rich stroma, its deletion also leads to low-stroma containing tumors that are more aggressive, have more undifferentiated histology, enhanced vascularity, and higher proliferation (Rhim et al., 2014). Further, a recent study has suggested that tumor-promoting behavior of the stroma when inhibiting the hedgehog pathway may be due to the increase in iCAF numbers and the immunosuppressive TME that is exhibited via the lower number of CD8^+^ T cells and higher number of regulatory T cells (Steele et al., 2021). Nevertheless, these observations illustrate the complex and multifaceted role of stroma in PDAC progression. 

Transforming growth factor β1 (TGF-β1)-regulated pathways also have a similar multifaceted effect on stroma-mediated PDAC progression as well as suppression (Tod et al., 2017). TGF-β1 regulates the highest number of overexpressed matrisome proteins in PDAC (Tian et al., 2019) hence, is a major driver of fibrosis. TGF-β is produced by stromal cells and its overexpression in the tissue microenvironment has been found in several precancerous disorders as well as in advanced tumors (Pickup et al., 2013) (Verrecchia, and Mauviel 2007) (Gordon, and Blobe 2008). Due to its crucial role in maintaining cellular homeostasis, TGF-β is expressed as an inactive precursor formed by the noncovalent association of mature TGF-β homodimer with the latency-associated peptide (LAP). LAP is encoded by the *TGFB1* gene, upstream to the region encoding mature TGF-β. Upon expression, LAP is processed from the N-terminal part of the TGF-β proprotein but remains linked to the precursor (Annes et al., 2003) (McGowan et al., 2003). LAP binds to the receptor-binding site on the mature TGF-β thereby hindering its binding to cell-surface receptors and inhibiting downstream signaling (Young and Murphy-Ullrich, 2004). Several stromal factors participate in the activation of TGF-β which involves the release of LAP from its receptor-binding site and thus facilitating a plethora of downstream signaling. Human PDAC samples with defective epithelial TGF-β signaling show higher epithelial STAT3 activity which results in the production of a rigid stroma, increased tension, and decreased patient survival (Laklai et al., 2016) (Winkler et al., 2020).

Integrins, as the principal receptors involved in cell-ECM interactions, adhesion, and mechanotransduction, are influenced by TGF-β signaling (Barczyk et al., 2010). Loss of TGF-β signaling and increased β1-integrin mechanosignaling in mice results in promoting tumor development by increasing matricellular fibrosis and tissue tension via STAT3 signaling (Laklai et al., 2016). On the other hand, epithelial STAT3 ablation slowed tumor development by decreasing stromal stiffness and epithelial contractility caused by TGF-β signaling loss (Laklai et al., 2016). Time course mass-spectrometry-based protein analysis of the PDAC cell lines incubated with CAF conditioned media showed an early activation of the MAPK pathway that was followed by overexpression of the STAT3 pathway and revealed a substantial enrichment of proliferation and EMT protein networks (Ligorio et al., 2019). Furthermore, a combined single-cell RNA sequencing of PDAC cells co-cultured with CAFs showed a significantly higher EMT and proliferation gene signature of the PDAC cells compared to when they were cultured alone. The gene signatures for proliferation and invasion of PDAC cells seem to be driven by CAF-secreted TGF-β (Ligorio et al., 2019). Collectively, these observations point out the complex and multifaceted roles of TGF-β signaling in tumor development and tumor-stroma interactions.

Studies have illustrated some differences between the ECM in the primary tumor and the tumor at the metastatic site. However, due to the low accessibility of metastatic tissue from patients and the lack of proper preclinical models, these studies are very limited. ECM components, including collagen and hyaluronan, are identified in high concentrations in both primary tumors and metastatic lesions according to a study analyzing patient samples from both primary and metastatic sites (Whatcott et al., 2015). No significant difference was seen in the extent of desmoplasia between primary tumors and metastatic lesions. Interestingly, solid stress is increased in liver metastases compared to the primary tumor for pancreatic cancer (Nia et al., 2017). However, this relationship is reversed for colorectal cancer and its liver metastases (Nia et al., 2017), demonstrating that mechanical abnormalities such as solid stress vary between tumors and are dependent on the cancer cell type and specific microenvironment. Despite efforts made to indicate the differences between tumor ECM at primary and metastatic sites, a comprehensive understanding remains elusive.

In summary, the desmoplastic reaction in PDAC has an important multifactorial yet incompletely understood role in disease progression and clinical outcome. To some extent, the non-linear correlation between the desmoplastic stroma and PDAC progression in part can be attributed to the contrary contributions from different subtypes of CAFs present in the tumor microenvironment.

## 3 Extracellular Matrix

The ECM appears in the body in two distinct types: basement membrane and interstitial matrix. The basement membrane is a thin layer of highly crosslinked specialized ECM proteins that is found in all tissues in the body and separates layers of epithelial or endothelial cells from the underlying connective tissue (LeBleu et al., 2007) (Jayadev, and Sherwood 2017). This membrane matrix maintains the polarization and functionality of the cells by retaining them in a permeable and loose structure (Karsdal 2019). Major components of the basement membrane include collagen type IV and laminin amongst others. The basement membrane owes its sheet-like architecture to two distinct polymeric structures which are formed by self-assembly of collagen type IV and laminin molecules, respectively. Although these collagen type IV and laminin networks are assembled independently, they are extensively linked by other ECM proteins such as nidogen and perlecan, increasing their mutual stability and ultimately providing structural integrity to the basement membrane (LeBleu et al., 2007) (Karsdal 2019) (Jayadev, and Sherwood 2017).

Laminin is the most abundant non-collagenous protein in the basement membrane that is essential for its proper organization and function (Rasmussen, and Karsdal 2019). Laminins are heterotrimer proteins, each containing one α chain, one β chain, and one γ chain (α1-5, β1-3, and γ1-3) which are linked in different combinations to comprise the 15 members of the laminin protein family (LeBleu et al., 2007). Laminins play important roles in the pathophysiology of cancer due to their influence on differentiation, adhesion, migration, and key steps in carcinogenesis (Maltseva, and Rodin 2018). Laminin subunit beta-3 (LAMB3) has been shown to be upregulated in the ECM of many malignancies including stomach, colon, lung, and pancreas (Tian et al., 2019) (Zhang et al., 2019). A recent study has shown that LAMB3 promotes invasion and metastasis in PDAC cells via the activation of the PI3K/Akt signaling pathway (Zhang et al., 2019). In contrast, another study has shown that culturing PDAC cell lines on laminin-coated plates slows down the cell migration compared to the uncoated, FN1 coated, and type I collagen-coated plates (Procacci et al., 2018). However, the type of laminin used in this study has not been indicated. Therefore, this apparent contradiction might be a result of using different laminin types.

The interstitial matrix, on the other hand, has proteoglycans and fibrous proteins as its two major macromolecule groups. Proteoglycans form a hydrated gel that fills most of the interstitial space within the tissue. The primary fibrous proteins in the interstitial matrix are fibrillar collagens (such as collagen type I and III), elastin, and fibronectins, which form long parallel polypeptide chains (Frantz et al., 2010) (Karsdal 2019). As with basement membranes, collagens are also the most common fibrous protein in the interstitial matrix (Frantz et al., 2010). Given the importance of different types of collagens, in constituting ECM and due to their significance in facilitating biochemical as well as biomechanical interactions in the PDAC tumors, they are discussed in detail in a subsequent subsection of this review.

The desmoplastic stroma of PDAC also shows an abundance of a large glycosaminoglycan called hyaluronan. Hyaluronan is a high molecular weight polymer synthesized by three isoforms of a membrane-bound enzyme, hyaluronan synthases (HAS1-3), located at the intracellular side of the plasma membrane (Liu et al., 2019). HAS isoforms catalyze hyaluronan synthesis in both stromal and carcinoma cells and high levels of these enzymes are linked to the accumulation of hyaluronan in the desmoplastic stroma (Auvinen et al., 2014) (Sato et al., 2016). Dysregulated hyaluronan buildup in the stroma severely affects its mechanical and biochemical characteristics and a hyaluronan-rich stroma is linked to poor prognosis in several carcinomas including PDAC (Amorim et al., 2021). In a recent study, pretreatment serum hyaluronan levels were found to be significantly higher in PDAC samples than in benign tumors or normal pancreata. It was also indicated that higher serum hyaluronan levels could be associated with metastasis and that this level could be used as a predictor of overall survival in PDAC patients (Chen et al., 2020). In ECM, hyaluronan and collagens interact in a complex manner that results in increased solid stress and interstitial fluid pressure (Provenzano et al., 2012) (Stylianopoulos et al., 2012) (Alexandrakis et al., 2004). ECM contains a range of hyaluronan polymers of different molecular weights, generated by enzymatic fragmentation of large hyaluronan polymers by hyaluronidase 1, 2, and 3 (HYAL1-3) and free radicals (Liu et al., 2019). Due to the diversity in their sizes, these fragments perform distinct functions in cell proliferation, migration, and differentiation. Hyaluronan molecules with high molecular weights can occupy the ECM in a way that leads to an increase in the compressive force in the TME resulting in reduced drug delivery (Sato et al., 2016).

Hyaluronan is primarily produced by fibroblasts present in the stroma and interacts with the cell surface receptor CD44 to regulate a cascade of pathways involved in cell proliferation, migration, and invasion, and thus plays an important role in cancer progression and metastasis (Bourguignon 2019) (Misra et al., 2015). CD44 is particularly important in pancreatic carcinogenesis since apart from being the principal cell-surface receptor for hyaluronan, it also provides anchorage for several other ECM components such as MMPs, collagens, laminin, and an important PDAC biomarker, osteopontin, all of which are upregulated in PDAC (Rychlíková et al., 2016) (Goodison et al., 1999). Incidentally, the binding of osteopontin to CD44 stimulates CD44 overexpression, providing additional binding sites for hyaluronan and other ligands and further aiding cancer progression (Marroquin et al., 2004).

Whilst 90% of the ECM proteins in malignant tumors are produced by stromal cells, such as fibroblasts, a fraction of the ECM-proteins originates from the cancer cells (Tian et al., 2019) (Naba et al., 2012). Many of these cancer-cell-derived ECM proteins promote tumor progression and metastasis in PDAC. Detection of elevated levels of these cancer cell-derived proteins in PDAC tumors has been frequently correlated with poor survival. These proteins regulate different cellular mechanisms to promote tumor growth and metastasis, thus leading to poor survival in preclinical PDAC models as well as in patients (Tian et al., 2019). On the other hand, some cancer-cell origin proteins (such as fibrillar collagens) have been shown to suppress tumor growth and metastasis (Tian et al., 2021). It is also interesting to note that these anti-tumorigenic properties were exclusive to cancer-cell-derived fibrillar collagens. Further, a recent study hints at the multifaceted characteristics of myCAFs in the tumor development showing that tumor-promoting myCAF-derived secreted factors can reverse the tumor-restricting effects of collagen type I produced by myCAFs in the stroma which complicates the stroma-targeting therapeutic strategies even further (Bhattacharjee et al., 2021). Another recent study, identifies two distinct subsets of pancreatic fibroblasts that are distinguished by the expression of CD105 (Hutton et al., 2021). It suggests that CD105^+^ pancreatic fibroblasts have tumor-promoting characteristics while their CD105^-^ counterparts have tumor-suppressive effects mediated with the functional adaptive immunity. Interestingly, both CD105^+^ and CD105^-^ cell populations could exhibit either iCAF and myCAF characteristics upon stimulation which challenges the previously thought paradigm of iCAFs being the pro-tumorigenic subtype and myCAFs being the anti-tumorigenic one.

Mass-spectrometry-based proteomics characterization of ECM in different pancreatic lesions and PDAC has shown that many overrepresented proteins in PDAC ECM are also overexpressed in several precursor lesions. For example, the cancer-cell derived matrisome proteins, AGRN, CSTB, and SERPINB5, which are overexpressed in PDAC stroma, are also expressed at elevated levels in early and late PanINs as compared to the normal pancreas (Tian et al., 2020), indicating that these proteins may have relevance in PDAC initiation and thus could potentially serve as early PDAC progression signatures (Tian et al., 2019).

Besides the chemical composition of ECM, its biophysical features also play a significant role in the pathophysiology of PDAC. Resident fibroblasts can significantly influence the organization and structure of stromal fibers, such as fibrillar collagens, by exerting tensile forces on the matrix and reorganizing collagen fibrils into sheets and cables, thereby influencing the ECM rigidity (Frantz et al., 2010). A higher proportion of randomly arranged ECM fibers were found in large and metastatic tumors, where randomly arranged ECM fibers were linked to poor prognosis. In contrast, parallel arrangement of ECM fibers in PDAC tumors was shown to be linked with better overall survival in patients (Bolm et al., 2020). However, contrary to the above report, another study by Drifka *et al.* suggested a more active role of stromal collagen topology in PDAC tumor progression. Based on the comparative structural analysis of stromal fibers in PDAC and non-malignant pancreatic manifestations using second-harmonic imaging microscopy, a unique spatial configuration of stroma was observed around the malignant ducts in PDAC which was characterized by increased alignment and cross-linking of collagen fibers (Drifka et al., 2015).

These diverse functions carried out by paralogous ECM proteins based on their cellular origin further signify the intricate nature of communication between cancer cells and the ECM.

### 3.1 Collagens and Pancreatic Ductal Adenocarcinoma

Collagens, being the major constituent of ECM, evidently play a crucial role in the interactions within the PDAC tumor microenvironment. They affect cell adhesion, migration, ECM remodeling, and EMT partly through the activation of their main class of receptors, discoidin domain receptor (DDR) family (reviewed elsewhere) (Orgel, and Madhurapantula 2019) (Huang et al., 2019). Collagens participate in different pathological conditions of the pancreas, including PDAC, and essentially involve the action of the major matrix processing enzyme, MMP. Collagens represent a large family of extracellular matrix molecules that play an important role in tissue assembly and maintenance. The collagen superfamily has 29 different known members (collagen type I - collagen type XXIX) in vertebrates (Kadler et al., 2007) (Gordon, and Hahn 2010) (Söderhäll et al., 2007). Being the principal structural components of the ECM, collagens contribute to a plethora of tissue functions, such as providing tensile strength, cell adhesion regulation, chemotaxis and migratory support, and tissue development directions. They also have a wide variety of roles in the functioning of the basement membrane (Kadler et al., 2007). Collagens, being secreted into the ECM by fibroblasts, are involved in cell-stromal interactions through different receptor families, the most common of which are integrins which also serve as receptors for laminins and fibronectin (FN1) (Nam et al., 2009). Most collagens are made of 3 α chains in either distinct (heterotrimers) or identical (homotrimer) combinations that fold together to form a triple helix. These triple-helical subunits systemically bind together to form collagen fibrils which cohere to form collagen fibers. Prior to the formation of the triple helix, collagens undergo substantial post-translational modifications which are mediated by several enzymes and molecular chaperones to ensure proper folding and trimerization of these proteins (Kadler et al., 2007).

The desmoplasia in PDAC stroma abundantly employs fibrillar collagens, such as collagens type I, III, and V as well as non-fibrillar collagens such as collagens type IV and VI. An *in vitro* wound-healing assay using PDAC cell lines also revealed the significance of collagen type I in cell migration and metastasis of PDAC cell lines *in vitro* compared to no coating and FN1 coating (Procacci et al., 2018). Collagen type I and collagen type III collectively account for approximately 90% of total collagen mass in different pancreatic pathologies and increase to nearly 2.6-fold during malignant transformation of the normal pancreas to PDAC (Tian et al., 2019). A recent study showed increased serum levels of collagen type III propeptide (PRO-C3) in patients with pancreatic malignancy. Moreover, the higher serum PRO-C3 levels resonated with more advanced stages of the disease and metastasis (Chen et al., 2020). Moreover, collagen type IV, which is an abundant network-forming collagen that accounts for nearly half of the basement membrane, is highly expressed and secreted by cancer cells. Being an important autocrine factor, it regulates the growth and migration of PDAC cells (Öhlund et al., 2013).

Besides the more abundant collagen types I, III, and IV, other collagen types, including collagen type VI, VIII, and XIV have also been shown to be upregulated in PDAC, as well as in chronic pancreatitis and PanIN, indicating common changes occurring in the stroma during the onset and progression of pancreatic malignancy (Tian et al., 2019). Collagen type VI has a beaded-filament structure and is of stromal origin (Tian et al., 2019). It forms a unique microfibrillar network in the basement membrane and interstitial matrix interface of many tissues (Sun et al., 2019). Apart from PDAC, collagen type VI is also upregulated in other malignancies, such as cancers of the colon, prostate, breast, and high-grade ovarian cancer amongst others (Xie et al., 2014) (Thorsen et al., 2008), where it promotes tumor progression, metastasis, and chemoresistance (Chen et al., 2013). Structurally, collagen type VI is a heterotrimer of a large tandem α-chain protein (*COL6A3*) and its two relatively smaller partners (*COL6A1* and *COL6A2*). The α3 chain of the collagen type VI protein (COL6A3) is particularly significant in the etiology of PDAC since the desmoplastic stroma in PDAC shows elevated levels of PDAC-specific isoforms of COL6A3. These PDAC-specific isoforms are a product of alternative splicing involving exon 3, 4 and 6, and are not detected in precursor lesions (Arafat et al., 2011). While PDAC-specific splicing of exons 3 and 6 of COL6A3 has been observed in 97% of the paired tumor-adjacent pancreas tissue samples, the presence of COL6A3 transcripts with alternate splicing of exon 4 is almost exclusive to the tumor tissue.

The importance of COL6A3 in different malignancies has been extensively studied. A microarray-based co-expression study using different gastrointestinal cell lines has shown that COL6A3 is co-expressed with a set of 62 genes, most of which are well-characterized oncogenes (Xie et al., 2014). At the core of this COL6A3 co-expression network lies FN1, which has been implicated for its role in cell adhesion, differentiation, and migration in many gastrointestinal tumors (Xie et al., 2014) (Pankov, and Yamada 2002). Another study, involving immunohistological comparison of a cohort of resected PDAC and adjacent pancreatic tissue, linked moderate to high levels of COL6A3 in PDAC tissue with negative prognostic factors, such as tumor differentiation, lymph node metastasis, perineural invasion, and microvascular invasion (Svoronos et al., 2020).

Apart from COL6A3, the α1 chain of collagen type VI (COL6A1) has also been shown to be involved in PDAC. A significant increase in COL6A1 expression was observed in a highly metastatic subset of cells harvested from the human pancreatic cancer cell line, BxPC-3 (Owusu-Ansah et al., 2019). Invasive and metastatic abilities of this invasive subset of cells decreased after *COL6A1* knockdown. Furthermore, retrospective IHC staining of paraffin-embedded PDAC tissue showed significantly higher levels of COL6A1 in cancerous tissue as compared to the para-cancerous regions. Also, increased expression of COL6A1 has been demonstrated as an independent predictor of decreased overall survival (Owusu-Ansah et al., 2019).

Collectively, collagens play a significant role in tumorigenesis and disease development owing to their interactions with different components of the stroma and their structural volume. They also provide a platform for other elements of the stroma to interact with each other, making them one of the most potential drug targets in regulating cancer.

### 3.2 Matrix Metalloproteinases

Molecular interactions within the ECM are tightly regulated and involve several matrix processing enzymes. MMPs, a group of zinc-dependent endopeptidases, significantly contribute to the regulation of stromal networking owing to their role in the catabolism of collagen and other ECM proteins (Knapinska et al., 2017) (Ricard-Blum 2011). Aggressive tumors recruit MMPs (especially membrane-bound MMPs, such as MT1-MMP) to remodel the tumor stroma and facilitate their invasion and metastasis (Jacob, and Prekeris 2015) (Nguyen et al., 2016). While MMPs are mostly secreted by stromal cells like inflammatory cells and fibroblast, they are also secreted by cancer cells (Kessenbrock et al., 2010). Due to their critical role in controlling tissue invasiveness, the expression of active MMPs in the tissue is tightly regulated. MMPs are synthesized as inactive zymogens and their activation is dictated by tight allosteric regulations (Sternlicht, and Werb 2001). Several cellular and ECM components influence MMP activation, where the furin-like proteinases are important players (Sternlicht, and Werb 2001).

Interestingly, *in vitro* studies on cultured PDAC cell lines showed that collagen type I significantly induce secretion and activation of MMPs, especially MMP-2 and MMP-9 in PDAC (Procacci et al., 2018). Both MMP-2 and MMP-9 are considered as important biomarkers for EMT in PDAC (Procacci et al., 2018). Retroactive analysis of a cohort of resected PDAC samples has also shown a strong correlation between elevated levels of MMP-8 and MMP-9, with poor patient survival (Hu et al., 2018). Although MMP-9 is overexpressed in PDAC (Qian et al., 2001) and has been suggested as a potential PDAC biomarker, its systemic depletion results in more invasive and metastatic tumors (Grünwald et al., 2016).

As mentioned above, MT1-MMP has been identified as a key modulator of desmoplastic reaction in pancreatic cancer progression since it is upregulated in PDAC and can directly activate TGF-β1 (Nguyen et al., 2016). By processing latent TGF-binding protein-1 (LTBP-1), MT1-MMP can also release latent TGF-β1 from the ECM (Tatti et al., 2008). Enhanced collagen synthesis by PDAC stellate cells as a result of MT1-MMP activation of TGF-β1 resulted in an increased fibrotic microenvironment (Krantz et al., 2011).

As mentioned previously, several stromal factors participate in the activation of TGF-β which involves the release of LAP from its receptor-binding site either mechanically or by proteolysis of LAP (Annes et al., 2003). Yu et al., showed that proteolytic cleavage of LAP by cell surface localized matrix metalloproteinases MMP-2 and MMP-9 releases mature TGF-β in the milieu. The secreted TGF-β effects downstream signaling in distant effector cells and can promote their proliferation and migration (Yu and Stamenkovic, 2000). Taken together, MMPs have an important role in the desmoplastic reaction as a result of their involvement in ECM turnover and in regulating critical signaling pathways.

## 4 Mechanical Traits

The desmoplastic reaction leads to stiffening of the stroma resulting in increased mechanical stress and rearrangement of collagen fibers within the ECM in a way that facilitates tumor progression and dissemination of cancer cells to metastatic sites (Freeman et al., 2017). Besides collagens, several factors contribute to make up of the mechanical microenvironment of PDAC tumors. Integrins, the predominant collagen receptors in the stroma, also modulate biophysical traits of the tumor through multiple mechanisms. The epithelium-specific integrin αvβ6 is upregulated during epithelial remodeling but is not expressed in healthy tissue. It interacts with a variety of ligands such as FN1, tenascin, vitronectin, LAP, and TGF-β3. Integrin αvβ6 is involved in the non-proteolytic activation of TGF-β by inducing conformational changes in the precursor complex which renders LAP unable to inhibit binding of TGF-β to its cell surface receptors (Annes et al., 2003). This mechanical activation of TGF-β is orchestrated by several stromal components (Munger et al., 1999). The secreted precursor LAP-TGF-β covalently associates with an ECM-glycoprotein, the latent TGF-β-binding protein (LTBP1) to form a tripartite complex also known as large latency complex (LLC). LTBP1 anchors itself in the ECM via fibronectin (Dallas et al., 2005). At the cell surface the transmembrane integrin αvβ6 binds to the LLC at the integrin-binding domain of LAP while its cytoplasmic domain binds to the actin cytoskeleton (Wipff and Hinz, 2008) (Annes et al., 2004). The mechanical stress in the stroma creates traction between ECM and the cells. This traction counters the strong non-covalent binding between LAP and TGF-β by inducing conformational changes in LAP and thus, results in the release of active or mature TGF-β (Wipff et al., 2007) (Robertson and Rifkin, 2016). Activation of TGF- β1 by integrin αvβ6 binding results in modulation of collagen fibril thickness. Integrins have also been shown to endorse invasion and metastasis in different carcinomas, including PDAC, partially by controlling the activity of MMP-2, MMP-9, and MMP-13 (Tod et al., 2017).

PDAC, like other solid tumors, shows a very high intratumoral interstitial pressure. The rapid proliferation of cancer cells together with angiogenesis increases the stromal pressure in the tumor which in turn causes tumor vessel leakiness and thus contributes to further increase the intratumoral interstitial pressure (McDonald and Baluk, 2002). The dynamics of these biomechanical events are further influenced by the outward interstitial fluid flow from the tumor core to its margins (Evje and Waldeland, 2019).

The term coined for this mutual relationship between these escalating mechanical forces within the tumor and their contributory factors is mechanoreciprocity, where tumor cells in the TME, when mechanically challenged, reciprocate by exerting a proportional cell-generated force (Paszek and Weaver, 2004). While this phenomenon pertains to the cell-generated forces at the single-cell level, it also considers the mechanical changes in the TME since each cell is constantly mechanically challenged by its surrounding microenvironment. The cell-generated mechanical forces originate at the cytoskeleton and are transmitted to the microenvironment via focal adhesion contacts on the cytoskeletal-integrin-ECM interface. This is carried out generally through myosin-dependent contractility that depends largely on the Rho-GTPase transduction cascade and Rho-Rock signaling (Shiu et al., 2004).

Rho-Rock signaling regulates motility and invasion of tumor cells by remodeling both the cytoskeleton as well as the tumor ECM (Rath et al., 2017). This is mainly achieved through TGF-β-induced EMT (Ungefroren et al., 2018). EMT involves a series of morphological changes that lead to motility and invasiveness of cancer cells, a prerequisite for metastasis (Ungefroren et al., 2018) (Safa 2020). The cytoskeleton-modulating activity of the Rho family of small GTPases involves regulation of actin filament nucleation, elongation, capping, and depolymerization (Lee and Dominguez, 2010). Reportedly, adhesion to the extracellular matrix in conjunction with the accompanying changes in cell shape and cytoskeletal tension is necessary for GTP-bound RhoA to activate its downstream effector, ROCK. Hence, it has been surmised that biophysical signals, such as cytoskeletal stress and adhesion maturation, and the intrinsic ROCK signaling form a feedback loop that contributes to a variety of mechanochemical activities in tumor tissue (Bhadriraju et al., 2007).

Different cells show varying degrees of sensitivity to biophysical changes depending on the tissue type. For example, neutrophils are sensitive to forces under 1 Pa while chondrocytes, which are constantly under mechanical load, show tolerance up to 20 MPa (Paszek, and Weaver 2004). It is interesting to note that normal epithelial cells, such as mammary cells, are sensitive to small changes in ECM stiffness and they reciprocally exert relatively small forces (Yeung et al., 2005). This contrasts with the strong response that is exhibited by mechanically active fibroblasts and cancerous mammary cells (Yeung et al., 2005). Limited data is available on biophysical sensitivity and cellular response in the pancreas and pancreatic tumors. However, PDAC tumors are known to exhibit a highly cross-linked desmoplastic reaction like what characterizes the mammary tumors and is considered as the main contributor to the latter’s high tensile strength and intratumoral pressure (Di Maggio, and El-Shakankery 2020).

In a study, Freeman et al. showed that the stress-induced invasion, which can be initiated by applying tensional loads on 3D collagen gel matrices in culture, is dependent on Rap1 GTPases. Rap1 GTPase belongs to the Ras-family of GTPases which has pleiotropic cellular functions including regulation of cell adhesion, proliferation, apoptosis, and cytoskeleton remodeling (Jaśkiewicz et al., 2018). Rap1 activity stimulates the formation of focal adhesion structures that align with the tensional axis (Freeman et al., 2017). Incidentally, Rap1-GTPases have also been implicated in EMT and metastasis in different cancer types (Freeman et al., 2010) (Zhang et al., 2017).

Mechanical characteristics of the tumors can be categorized into 4 major traits: solid stress, interstitial fluid pressure, stiffness, and tissue microarchitecture (Nia et al., 2020). While the former two are extrinsic by nature and depend on environmental cues, tissue stiffness and microarchitecture are intrinsic properties of the tissue. The degree of invasiveness of tumor cells is determined by an interplay between different mechanical traits exhibited by the tumor (Nguyen et al., 2016).

Solid stress is generated due to the excessive number of proliferating cells that apply chronic compressive stress on the neighboring tissues. Elevated solid stress within tumors triggers the expression of Growth Differentiation Factor 15 (GDF15). GDF15 is a TGF-β-family ligand whose cellular expression also takes cues from other prevailing cellular stresses such as hypoxia and nutritional stress (Patel et al., 2019) (Wang et al., 2021). GDF15 activates the Akt-signaling pathway in the cancer cells leading to invasiveness, and migration (Kalli et al., 2019) (Nia et al., 2020). On the other hand, it has been shown that solid stress suppresses tumor growth in spheroids independent of the tissue of origin, host species, or differentiation status (Helmlinger et al., 1997). An earlier study measured this suppressive aspect of solid stress within the tumor to be 45–120 mm Hg (∼6–16 kPa) which is substantially higher than the blood pressure in the tumor vasculature and is sufficient to cause the collapse of the vasculature which explains the impaired blood flow to the tumor (Helmlinger et al., 1997). Another study measured the compressive solid stress exerted by tumor cells to be as high as 3.8 kPa (∼30 mm Hg) in the center of murine pancreatic tumors (Nia et al., 2017) while the measured solid stress in normal soft tissues such as kidney and liver was negligible (Nia et al., 2017). These studies have emphasized that solid stress or the intrinsic intertumoral stress due to accelerated cell proliferation, is a fundamental aspect of cancerous tumors that severely affects the tumor vasculature and as a result the intertumoral drug distribution.

The interstitial pressure within tumors depends on extrinsic factors and is controlled by circulatory systems. It has been shown that compression of lymphatic vessels increases interstitial fluid pressure while blood vessel compression decreases blood flow and consequently the interstitial pressure. The core of nearly all solid tumors exhibits chronically elevated levels of interstitial pressure that abruptly decreases to almost zero around the edge of the tumor creating a radial fluid flow towards the neighboring tissue that provides an escape route for disseminating cancer cells and consequently leads to invasion and metastasis via flow-induced shear stress (Nia et al., 2020). In addition, reduced blood flow because of high solid stress results in a hypoxic TME. Tumor cells residing in this hypoxic microenvironment are forced to switch their metabolic activity from oxidative phosphorylation to glycolysis (the Warburg effect) in order to survive (Hanahan, and Weinberg 2000). This metabolic change, together with other contributing factors results in tumor progression, immunosuppression, inflammation, and eventually invasion and metastasis. These changes also render most anti-cancer therapies ineffective (Stylianopoulos et al., 2012).

Increased stiffness is another mechanical alteration that is developed in tumors and has been observed in many solid tumor types including pancreatic cancer (Nia et al., 2020). Matrix stiffening is mainly caused by increased matrix deposition rates and crosslinking of collagens. Moreover, high levels of tensile stress in the microenvironment can also lead to increased stiffness through “strain stiffening”, a phenomenon representing non-linear changes in the elasticity of collagen fibrils (Storm et al., 2005). Higher stiffness activates proliferative, invasive, and metastatic signaling pathways within the tumor cells and is correlated with chemoresistance (Deville, and Cordes 2019). Recent studies have proposed using tissue stiffness as a diagnostic marker and predictor of poor prognosis (Nia et al., 2020).

Microarchitecture in normal tissue determines homeostasis and provides signals for the transformation of the tissue. High level of proliferation, matrix deposition, and increased stiffness in tumor tissue results in altered microarchitecture that alters the interactions between individual cells in the TME and their surrounding matrix. This may result in the reprogramming of the cells, which in turn will activate signaling pathways associated with invasion and metastasis (Mayer et al., 2018) (Nia et al., 2020).

Interestingly, despite having the same stiffness, pancreatic primary tumors have higher solid stress compared to their derivative metastatic counterparts in the liver showing that stiffness and solid stress are two distinct mechanical anomalies in tumors that should be targeted separately (Nia et al., 2017). Since carcinogenesis is linked to substantial changes in tissue tension as well as modifications in force sensing by transformed cells, it seems very likely that altered mechanotransduction and the loss of tensional homeostasis are important factors in epithelial tumor pathogenesis (Paszek and Weaver, 2004). Collectively, these observations strongly correlate PDAC progression and invasiveness with substantial changes in the biophysical traits of the tumor tissue.

## 5 Models to Study Interactions With Extracellular Matrix and Tension/Pressure

It is clear, then, that the effects of the ECM on tumor progression are simultaneously multifaceted, operating at multiple levels of analysis (physically and chemically), and are often both instructive and consequential with respect to disease progression. In order to disentangle the biomechanical and biochemical roles that the ECM plays in PDAC, effective tools and models are required. Avenues to explore the effect of tissue tension are relatively limited with traditional, two-dimensional, cell culture, however, tools are available such as culture on elasticated tissue culture surfaces that can be put under uni- or bi-directional tension (Kamble et al., 2016). Although the direct application of these devices in the context of PDAC does not appear to have been performed, such devices have been used to identify that breast cancer cells undergo increased proliferation and migration in response to mechanical strain and that exosomes derived from these breast cancer cells induce an immunosuppressive phenotype (Wang et al., 2020). Alteration in tumor cell migration has also been identified in other tumor cell lines via induction of Rap1 activity both in two- and three-dimensional (3D) cultures (Freeman et al., 2017). Critically, two-dimensional models result in monolayer cell cultures, where cell morphology is altered because of the format, inducing artefactual pressures on the cell, such as a forced polarity and a flatted nucleus which can alter cell signaling, compared to a 3D counterpart (reviewed elsewhere) (Yamada and Cukierman, 2007) (Kim 2005) (Kapałczyńska et al., 2018).

Recently, 3D organoid cultures of primary cell lines derived from a variety of organs have been developed (Barker et al., 2010) (Sato et al., 2011) (Huch et al., 2015) (Rosenbluth et al., 2020), including those of the pancreatic ductal epithelium and their tumor counterparts (Boj et al., 2015) (Baker et al., 2016). A contemporary review comprehensively explores developments of PDAC culture models for broad application (Heinrich et al., 2021), however, typically PDAC organoid cultures are maintained in a 3D hydrogel-based scaffold comprised of a variety of basement membrane proteins such as Matrigel (Baker et al., 2016). Matrigel is derived from basement membrane extracts of murine Engelbreth-Holm-Swarm sarcoma, and contains laminin, collagen type IV, and entactin (Kleinman and Martin, 2005).

One advantage of 3D culture in hydrogel scaffolds is that they are biomechanically more representative of tissue than traditional two-dimensional cultures on plastic and permits examination of cell interactions with a pre-established matrix (Öhlund et al., 2014). Here we present some examples of hydrogel composition and construction, however, a comprehensive examination on hydrogel construction and developments is well beyond the scope of this review but is deftly addressed elsewhere (Karoyo and Wilson, 2021) (Zhu and Marchant, 2011) (Caliari and Burdick, 2016) (Gyles et al., 2017). Hydrogels are highly customizable in composition and can be formed from a variety of substrate polymers both natural and synthetic as well as a differential contribution to chemical cell signaling. Alginate and polyethylene glycol, for instance, are relatively inert whereas collagens, laminin, and/or FN1 can contribute directly to cell signaling (Miroshnikova et al., 2011) (Caliari and Burdick, 2016). This ability to tailor matrix composition can be utilized to assist in decoupling the mechanical and biochemical signaling effects of the ECM on the growth of tumor organoids (Chang and Lin, 2021). Indeed, seaweed-derived alginate, which lacks the native ligands that permit biological signaling with mammalian cells can be utilized to alter matrix mechanics without contributing to biological matrix signaling (Rowley et al., 1999). Further, alginate has been used in an *in vitro* breast cancer model where fine-tuning the elasticity of the gel was sufficient to determine an inverse correlation between tumor cell proliferation and tissue hardness (Cavo et al., 2016).

Hydrogel stiffness is dependent on several factors such as the polymer used, concentration, and cross-linking method. Stowers *et al.* developed an alginate-based system of tunable stiffness that is dependent upon the concentration of ionic calcium chelators that promote polymer cross-linking (Stowers et al., 2015). The concentration of calcium available could also be modulated photo-dynamically, thus allowing characterization of cell cultures over time under altering matrix stiffness. In this example, the migration mode of fibroblasts altered in response to differing matrix stiffness, with an inverse relationship between matrix stiffness and cell elongation and migration (Stowers et al., 2015). Alternatively, synthetic polyacrylamide (PA) gel stiffness can be modified by the concentration of the monomer, cross-linker, and the temperature of gelation, permitting robust and reproducible investigation of matrix stiffness on cultured cells (Denisin, and Pruitt 2016). Further, there are several examples where hydrogels composed of the synthetic polymer polyethylene glycol (PEG) have their stiffness modulated photodynamically, which offers the opportunity to examine the effect of inducing ECM stiffness on cellular behavior (Mabry et al., 2015) (Liu et al., 2018) (Günay et al., 2019) (Kalayci et al., 2020).

Regardless of how the matrix is constructed *in vitro*, effective quantification of its mechanical and biochemical properties is essential for hypothesis testing. With respect to biochemical properties, composition and alteration can be readily examined by means of immunohistochemistry and immunofluorescence, or destructively by means such as mass spectrometry. Additionally, histological analysis by Masson’s trichrome and picrosirius red can be used to examine collagen structure, linearization, and orientation. Further, dynamic techniques that can acquire such data over time can be applied to live cultures and tissue explants and thus capture complex spatiotemporal dynamics of matrix remodeling. Such techniques include second harmonic generation (SHG) that can also be used to examine collagen fiber alignment (Fuentes-Corona et al., 2019).

In contrast, when it comes to measuring the mechanical properties of the ECM there are several methods to measure the mechanical properties from the cellular to tissue scales. Techniques such as atomic force microscopy (AFM) can measure sample stiffness on tissue down to the nanometer range and have demonstrated utility as a diagnostic tool in other cancer types such as breast and liver (Stylianou et al., 2018) (Tian et al., 2015) (Plodinec et al., 2012) (Rother et al., 2014). Additionally, tensile and compression testing can be used to assess the viscoelastic properties of soft tissue samples, and so can rheometry for fluid samples (Buckley et al., 2009) (Griffin et al., 2016). In addition, techniques such as mesoscale indentation are well suited for the examination of nuanced regional mechanical heterogeneity in tissue (Rubiano et al., 2018). This approach applied to human healthy and PDAC pancreatic samples identified that PDAC tissue exhibited a higher steady-state modulus than normal tissue on average (Rubiano et al., 2018). Furthermore, isolated tumor-associated stromal cells cultured in collagen hydrogels formed stiffer hydrogels when exposed to conditioned medium from PDAC cells than when in control medium (Rubiano et al., 2018).

There exist a range of tools by which the effect of matrix properties on PDAC behavior can be examined and the majority of consideration in the literature on this topic has concentrated on the effects of biochemical matrix composition (Biondani et al., 2018) (Begum et al., 2017) (Tian et al., 2019). In contrast, comparatively little has been elucidated on how the physical properties of the matrix affect PDAC behavior. Whilst *in vivo* murine models have determined that though alleviating solid stress within the tumor by means of removing the dense stromal tissue of the tumor may improve drug perfusion to the tumor site, doing so also leads to increased invasiveness of the cancer cells which are no longer constrained by the surrounding tissue (Rhim et al., 2014). However, in this example, the role of physical properties is not disentangled from the other signaling of the stromal cells present. Experiments culturing established PDAC cell lines in polyacrylamide gels of differing stiffnesses (1 kPa, 4 kPa, and 25 kPa) identified that vimentin expression and nuclear β-catenin localization, both markers for EMT, increased with matrix stiffness (Rice et al., 2017). Of therapeutic relevance, these same experiments demonstrated that matrix stiffness promoted resistance of the cells to paclitaxel, however, cells remained sensitive to gemcitabine regardless of stiffness (Rice et al., 2017). The role of cell stiffness in PDAC invasion potential has also been explored by models utilizing cell lines in both traditional scratch wound healing and transwell migration assays which identified that stiffer pancreatic cell lines tend to be more invasive (Nguyen et al., 2016).

The effect of matrix stiffness on tumor cells does not yield a comprehensive understanding of the effects of the mechanical properties of the matrix on overall tumor development as CAFs, for example, are a non-malignant cell type that are critical in modulating tissue stiffness. CAF matrix production is influenced by matrix pressure, whereby CAFs under pathological tissue stiffnesses (∼7 kPa) produce a matrix resembling pathological ECM (Malik et al., 2019). However, when cultured under normal physiological stiffnesses in polyacrylamide gel (∼1.5 kPa) CAFs generate an ECM that is similar to that of normal fibroblasts (Malik et al., 2019). Further, the culture of human CAFs in 3D conditions identified that the mechanical quiescence of PSCs can be restored by means of all-trans retinoic acid (ATRA) in addition to the culture medium (Chronopoulos et al., 2016). Additionally, pancreatic cancer cells readily migrated into matrices remodeled by control PSCs but demonstrated minimal invasion into matrices remodeled by ATRA-treated PSCs (Chronopoulos et al., 2016). An alternative study also demonstrated that modulation of ECM stiffness itself was a key factor in PSC activation to CAFs by means of culture on polyacrylamide gels of varying stiffnesses (from 1 kPA–25 kPA), where high stiffness matrices induced a myofibroblastic-like phenotype in the PSCs (Lachowski et al., 2017). In this example, it should be observed that 25 kPa is much stiffer than typical for the upper quartile range for PDAC of 4 kPa, although stiffnesses of up to 10 kPa are observed (Rice et al., 2017). An alternative examination of PSC density and matrix stiffness identified that increased PSC cell density increased tissue stiffness up to 1 kPa (Robinson et al., 2016). Further, it was identified that PSC-mediated re-modeling of the collagen matrix was dependent on the cellular contractile apparatus of the PSCs since when actomyosin contraction was blocked by means of blebbistatin, collagen fiber alignment was severely inhibited compared to the untreated control (Robinson et al., 2016). Similar results were also identified when PSCs were treated with ATRA, inhibiting their activation, indicating again the requirement of PSC activation for matrix remodeling (Robinson et al., 2016).

Overall, the field is ripe for addressing the question of the mechanical influences of the matrix on PDAC. The development of robust and relevant 3D organoid models of PDAC, coupled with the availability of tunable matrices both in terms of stiffness and composition provides a great opportunity for exploration. Furthermore, the development of 3D *in vitro* co-culture models of PDAC whereby PDAC tumor organoids are cultured together with PSCs leads to the formation of multiple CAF subtypes and allows for the examination of the signaling loops between these various cell types (Öhlund et al., 2017). These models could similarly be utilized to examine the interplay between CAFs, tumor cells, and their matrix context, for example by culture in matrices of differing initial stiffness. Further, as CAFs are themselves a key modulator of tissue stiffness, examination of co-culture models could provide clues as to how to interfere with CAF-mediated modulation of matrix stiffness. Rubiano *et al.* have worked on generating an *in vitro* model of PDAC that mimics the pathology with respect to the stiffness of PDAC tumors in conjunction with PDAC cells co-cultured in collagen gels with patient-matched PSCs (Rubiano et al., 2018). Whilst this model does not use organoids, it would be encouraging to explore whether this work could be adapted to this end.

## 6 Clinical Implications

Traditional diagnosis and treatment strategies against PDAC target cancer cells and cancer cell-derived biomarkers. However, since desmoplastic reaction accounts for up to 80% of the tumor volume (Erkan et al., 2012), characterizing PDAC tumor stromal components for identifying potential biomarkers and drug targets would not only improve the chances for early detection but also enhance the effectiveness of current treatment strategies aimed against advanced stages of the disease. As explained above, desmoplasia causes excessive extracellular matrix turnover, which includes constant breakdown and biosynthesis of collagens resulting in the concurrent discharge of collagen fragments into circulation (Willumsen et al., 2019). Nevertheless, desmoplasia is one of the main culprits in failed systemic treatments against PDAC. It not only hampers drug delivery, owing to its role in increasing pressure and decreasing circulation within the tumor, but also contributes in treatment resistance through its influence on intracellular signaling and biomechanical properties of the tumors (Saini et al., 2018). In this section, we review the current knowledge and discussions around the clinical implications of stromal contents in PDAC and the possibilities of establishing them as potential drug targets for future treatment strategies.

### 6.1 Diagnostic Implications

Conventional tumor-derived biomarkers, such as CA 19-9, TPS, CEA, and Ca 125 together with circulatory tumor cells, have long been used for diagnosis, monitoring disease progression, and prognosis in PDAC (Franklin et al., 2015) (Martini et al., 2019). However, owing to their origin from cancer cells, these biomarkers usually appear in circulation during advanced stages of the disease leading to late diagnosis, one of the hallmarks of PDAC, and limiting the scope of a successful treatment regimen. In recent years, measuring circulating ECM components in the blood has emerged as a new strategy to identify a novel source of potential biomarkers which can be used in the early detection of the disease (Franklin et al., 2015). This strategy holds an immense potential since the detection of differentially expressed levels of stromal cell-derived proteins and other biomolecules, pertaining to the earlier stages of the disease, could lead to a better diagnosis and, in turn, prognosis of the disease. Moreover, detection of stroma-derived biomarkers from precancerous lesions could pave the way for establishing a successful preventive medicine system for PDAC, thus further signifying the importance of studying stromal-derived ECM components (Tian et al., 2019). CAFs, being the main contributors to desmoplasia, can detect and react to cues from cancer cells even from early stages and provide an opportunity for early detection. Many groups have studied the precision of different stromal components and their specificity in diagnosis and predicting patient outcomes. Collagens, being the main components of the ECM, have justifiably garnered considerable attention from PDAC researchers. Since collagens in the desmoplastic ECM are constantly being produced and degraded, measuring both intact and degraded collagen-derived epitopes in the circulation could be used to determine the collagen as well as ECM turnover in the pancreas. Deviations from the carefully maintained ECM homeostasis of healthy stroma could indicate an impending anomaly such as the onset of PDAC (Willumsen et al., 2019).

As the main fibrous proteins of the tissue, collagens type I and III have been analyzed often. In a phase-III clinical study, measured pre-treatment serum levels of several different MMP-degraded collagens including type I (C1M), type III (C3M), type IV (C4M), and pro-peptide of type III (PRO-C3), in patients with stage III or IV PDAC were found to be above the reference range for 67–98% of patients with median values being almost two-fold as compared to the healthy controls (Willumsen et al., 2019). High levels of all the collagen fragments were associated with shorter survival while a higher degradation/formation ratio of collagen type III (C3M/PRO-C3) was associated with better overall survival (Willumsen et al., 2019). It can be surmised that a similar patient-sample-based approach for detecting levels of circulating-collagen turnover, that corresponds to specific stages of disease progression, could help in establishing a credible circulatory collagen-based tool for monitoring progression of PDAC and/or response to treatment.

As a result of the distinctive structure and role of collagen type VI in the ECM, researchers have often investigated it as a potential biomarker for several metabolic diseases including PDAC (Sun et al., 2019). Retrospective analysis of serum samples of patients has shown significantly higher levels of COL6A3 protein in the serum of patients with PDAC compared to serum from healthy subjects or patients with benign lesions (IPMN and cystic lesions) (Kang et al., 2014). The elevated level of COL6A3 did not have a significant association with overall survival but it was able to predict survival better than the CA19-9 level, a carbohydrate biomarker that is currently the most frequently used biomarker to monitor tumor progression in PDAC patients (Kang et al., 2014). A recent study, analyzing different fragments of collagen type VI, found elevated levels of MMP-cleaved fragments of COL6A1 and COL6A3 in serum of patients with different stages of solid tumors, including PDAC, than in the serum obtained from subjects with either a healthy or non-cancer pancreas. This suggested that MMP-mediated collagen type VI alteration is crucial in carcinogenesis and that measuring collagen type VI fragments in serum might be used as a tumor biomarker (Willumsen et al., 2019). Although endotrophin, a collagen type VI-derived peptide, was previously measured to be elevated in PDAC (Sun et al., 2017), it did not show any significant difference in this study (Willumsen et al., 2019).

While individual stromal components such as collagens and their cleavage products, are widely accepted as potential biomarkers, most preclinical approaches and clinical trials have emphasized the importance of using diagnosis panels with a combination of different biomarkers that include both stromal and tumor-derived markers in order to have a holistic view of the disease characteristics in patients (Balasenthil et al., 2017). A recent study demonstrated this notion by showing that when tested using a panel of eight biomarkers, including both tumor and stroma-derived biomarkers, some patient samples presented with elevated levels in only one or two biomarkers which in few cases were only stroma-derived biomarkers (Franklin et al., 2015).

### 6.2 Therapeutic Implications

Different components of the stroma have also been studied to find new therapeutic opportunities. Many anti-desmoplastic agents have been used to target these components. These treatment options either target the mechanical properties of the stroma, such as high interstitial pressure and solid stress, or target specific molecular components regulating tumorigenesis, such as TGF-β, Sonic Hedgehog. Recently, Neesse et al., have extensively reviewed the clinical significance of biophysical and molecular aspects of PDAC stroma (Neesse et al., 2019). Here, we highlighted selected stroma-associated targets that have been used in clinical trials or had promising preclinical results.

#### 6.2.1 Targeting the Mechanical Properties of the Tumor

Recent decades have seen more focused research on developing treatments that target different mechanical traits of tumors. As mentioned previously, four major mechanical characteristics of tissue that are altered during malignancy include solid stress, interstitial fluid pressure, stiffness, and tissue microarchitecture. Although treatments targeting stiffness and solid stress in tumors have been previously explored, fewer studies have targeted interstitial fluid pressure and altered microarchitecture of a tumor.

The main influencers of the increased stiffness in the tumor tissue are the high levels of matrix deposition in the desmoplastic stroma and increased matrix cross-linking (Nia et al., 2020). Moreover, since solid stress is mainly exhibited in ECM, many of its adverse physical effects such as compressed vasculature, can be reversed by relieving the stress in ECM (Nia et al., 2020). Therapeutic agents that primarily act by selective depletion of one or more of the abundant ECM components often reduce the total stromal volume of the tumor. This, in turn, decreases the solid pressure and stiffness and alleviates compression on the vasculature, allowing increased intratumoral circulation and perfusion and thus improve drug delivery to the tumor. Most therapeutic strategies to counter solid stress in the tumor involve individual or combined targeting of cancer cells, CAFs, collagen, or hyaluronan (Figure 2).

As the major component of the ECM, collagen types I, II, and III have been the focus of several clinical trials. These trials include drugs that neutralize collagens or accelerate their degradation, such as anti-inflammatory drugs or collagen-neutralizing antibodies (Choi et al., 2013) those which interfere with collagen expression directly, such as micro-RNA based drug MiR-129-5p (Wang, and Yu 2018), or those which target enzymes regulating collagen biosynthesis and stability, such as 4-hydroxylase which is involved in stabilizing the collagen triple helix (Gorres, and Raines 2010) (Vasta, and Raines 2018). Conventional multi-drug regimens which use chemotherapeutic agents such as nab-paclitaxel and gemcitabine have also been shown to affect collagen content of the ECM. Tumor samples from patients treated with this combination-treatment showed decreased collagen type I content coupled with disorganized collagen networks and fewer CAFs compared to untreated samples (Alvarez et al., 2013).

An important class of tumorigenic components of desmoplastic stroma is MMPs, which regulate collagen metabolism. Despite several reports linking the MMPs with PDAC carcinogenesis and evidence of the effect of their inhibition in survival (Bramhall et al., 2001), their broad-based inhibition in PDAC did not yield encouraging results in clinical trials mainly due to adverse side effects such as musculoskeletal toxicities (Bramhall et al., 2002). Most of these drugs showed a very low therapeutic index, where both higher and lower doses induced toxicity (Peterson 2006). This suggests the significance of maintaining equilibrium in MMPs’ activity for tissue homeostasis. It has also been surmised that the success of any MMP inhibitor would also greatly depend on the stage of the disease at the time of commencing the drug regimen since both premature and late delivery of the drug would result in adverse results, thus leaving a very narrow therapeutic window corresponding to a specific premetastatic stage of the tumor (Knapinska et al., 2017). Nevertheless, rapidly accumulating evidence on the role of aberrant MMP activity in different aspects of PDAC progression and metastasis has necessitated the need for reassessment of strategies targeting these MMPs.

Another component of the ECM that contributes significantly to stromal stiffness is hyaluronan. Combined biochemistry of hyaluronan and collagens in the TME has been shown to increase solid stress in the tumors (Stylianopoulos et al., 2012). One study has shown that collagens and hyaluronan work in tandem to compress intratumoral vessels. An angiotensin inhibitor, Losartan, which acts by reducing the production of hyaluronan and collagen in CAFs, has been found effective in reducing solid stress and improving drug and oxygen delivery in PDAC tumors (Chauhan et al., 2013). The activity of PEGylated human recombinant PH20 hyaluronidase (PEGPH20), a hyaluronan degrading enzyme developed as an anticancer drug, has also been found to enhance doxorubicin and gemcitabine chemopermeability. A combination drug regimen that used PEGPH20 in conjunction with gemcitabine in a preclinical mouse model resulted in suppression of pancreatic tumor development and better survival compared to gemcitabine alone (Jacobetz et al., 2013), however, results from clinical trials have not been conclusive (Hingorani et al., 2018) (Van Cutsem et al., 2020). The phase I/II study (NCT01839487) resulted in better prognosis for patients who had high pre-treatment levels of hyaluronan. However, the phase III study (NCT02715804) was stopped due to the ineffectiveness of the combined PEGPH20 with gemcitabine and nab-paclitaxel to improve the overall survival, progression-free survival, and the duration of response compared to gemcitabine and nab-paxitacel alone which resulted in the withdrawal of interest in the drug by the sponsor company (Tucker 2019) (Halozyme Therapeutics 2019). Another strategy to reduce desmoplasia and remove excess ECM is to target the cells producing ECM, namely CAFs. However, this strategy has presented challenges as well. Several novel and repurposed small-molecule drugs have been tested for their anti-desmoplasia effects in preclinical as well as clinical trials (Nandi et al., 2020). Some of the most intriguing, repurposed candidate drugs include Aspirin, Metformin and Chloroquine which are tested for their anti-desmoplasia activity by targeting mainly CAFs, pancreatic stellate cells (PSCs) and cancer stem cells (CSCs), respectively (Takada et al., 2004) (Incio et al., 2015) (Balic et al., 2014).

Despite encouraging results at the pre-clinical stage, many promising anti-desmoplasia drug candidates targeting either CAFs or other stromal components, fail to give conclusive results in clinical trials, a trend they share with many anti-cancer drugs. The main reason for this chemoresistance being desmoplasia itself. To circumvent this issue, in a recent study Nicolásnicolás-Boluda *et al.* tried to deplete CAFs in cholangiocarcinoma by using gold nanoparticle-mediated photothermal therapy and successfully decreased tumor stiffness which subsequently resulted in tumor regression (Nicolásnicolás-Boluda et al., 2020). Similar unconventional approaches are also required to negate the effect of desmoplastic reaction in PDAC and normalize the tumor mechanics to increase the effectiveness of both conventional and novel drug therapies.

#### 6.2.2 Targeting Tumor-Promoting Stromal Components

Depletion of desmoplastic reaction in stroma has been found effective in eliciting initial drug response in tumors, however, targeting abundant non-neoplastic tumorigenic components of the stroma, such as CAFs, CSCs, and regulatory T cells, is required for effective and long-lasting treatment strategies and better prognosis. Targeting the tumorigenic components in TME, especially CAFs, has shown encouraging results for reducing relapse and metastasis (Sunami et al., 2021). One of the most significant events in tumorigenesis is the activation of quiescent PSCs to form CAFs (Apte et al., 2012). The process is governed by the cancer cells and involves several signaling pathways, such as hedgehog signaling and TGF-β signaling, as well as specific environmental cues, such as hypoxia and malnutrition. Many of these CAF-activation factors have been exploited by researchers to find efficient anti-cancer therapies (Wu et al., 2021).

Patients with PDAC present with deficiencies in fat-soluble vitamins such as A, D, and K which mainly stem from exocrine pancreatic insufficiency (Min et al., 2018). Vitamin A plays a key role in maintaining pancreatic homeostasis and its deficiency leads to the activation of PSCs. In a preclinical study, Froeling *et al.*, exposed activated PSCs to a vitamin A homolog, ATRA. Incubation with ATRA resulted in altered gene expression in PSCs which caused their phenotype to revert to the quiescent state and suppressed genes involved in proliferation and motility (Froeling et al., 2011). ATRA has also been repurposed as a stromal targeting agent to be used in a phase-I clinical trial and after being declared safe to use in combination with gemcitabine-nab-paclitaxel it has been approved for phase II clinical trials (Kocher et al., 2020). Likewise, vitamin D receptor has been identified to act as a master transcriptional regulator for PSC quiescence, suggesting that priming the pancreas with calcipotriol (a vitamin D analog) could support PDAC therapy (Sherman et al., 2014). Tests in murine models of PDAC where gemcitabine treatment was supplemented with calcipotriol showed reduced stromal activation, together with an increased intratumoral concentration of gemcitabine and longer median survival. Further, since paricalcitol (another vitamin D analog) has been demonstrated to be effective in a pre-clinical setting (Schwartz et al., 2008), it is currently being tested in several clinical trials (NCT03331562) (NCT03415854) (NCT03520790) (Sherman et al., 2014). However, an earlier clinical trial on patients who had their treatment regimen supplemented with seocalcitol (another vitamin D analog) failed to show an objective response to treatment (Evans et al., 2002). With respect to vitamin K, an inverse association between dietary intake of phylloquinone (vitamin K1), but not menaquinones (vitamin K2), and risk of pancreatic cancer has been recently identified, however little seems to be known about this association at the molecular level (Yu et al., 2021).

In addition to environmental cues, cellular communication between cancer cells and quiescent PSCs plays an important role in stromal remodeling during PDAC tumorigenesis(Apte et al., 2012). Important paracrine signals from cancer cells include hedgehog signaling ligands such as SHH which bind to its receptor Patched1 (PTCH1) on the PSCs and via uncoupling of SMO, activates the signaling cascade (Saini et al., 2019). SHH is a key development regulator which is absent in the normal pancreas but has been shown to be overexpressed in PDAC (Kayed et al., 2006). SHH-mediated hedgehog signaling plays an important role in regulating differentiation of PSCs and proliferation and motility of CAFs and is considered an important tumorigenic factor (Bailey et al., 2008). Immuno-blocking of SHH by monoclonal antibody 5E1 has led to the depletion of desmoplasia and formation of smaller tumors in a PDAC mouse model (Bailey et al., 2008). Similar effects were observed when SMO was inhibited on PSCs with cyclopamine, a naturally occurring alkaloid and inhibitor of SHH signaling (Li et al., 2014) (Ma et al., 2013).

Another core signaling pathway involved in PDAC tumorigenesis is TGF-β signaling. Downregulation of TGF-β signaling in PSCs is considered a crucial molecular event during PDAC progression owing to its tumor-suppressive effect (Neuzillet et al., 2014). On the other hand, TGF-β signaling also regulates PSC activation, and TGF-β activated CAFs have been implicated in tumor growth and metastasis (Calon et al., 2014). Despite the role of regulatory T cells (Treg) in immune suppression, their depletion in a PDAC mouse model led to the unexpected result of accelerated tumor progression due to their ability to reprogram the fibroblast population and deplete αSMA^+^ tumor suppressive CAFs via the loss of TGF-β ligands (Zhang et al., 2020). Regardless of its dual role in PDAC tumorigenesis, several small-molecule TGF-β signaling blockers such as Vactosertib (TGF-β type 1 receptor kinase inhibitor) and Luspatercept (allosteric inhibitor of TGF- β ligand) have been shown effective in targeting PDAC progression and metastasis (Kim et al., 2021). However, inadvertent side effects, such as inhibition of other tumor-suppressive pathways, have limited clinical applications of many TGF-β signaling antagonists (Ciardiello et al., 2020).

Another key event in PDAC tumorigenesis is angiogenesis which is driven by an intricate balance of several pro-angiogenic factors, such as vascular endothelial growth factor (VEGF), fibroblast growth factors, epidermal growth factor, in antagonism with anti-angiogenic factors, such as angiostatin and endostatin. Several anti-angiogenic drugs have been used to target angiogenesis in PDAC. Most of these drugs such as Axitinib, Bevacizumab and Nintedanib (Kindler et al., 2011) (Bill et al., 2015) (Crane et al., 2009), target VEGF and cause depletion of the tumor stroma followed by decreased tumor growth (Bergers, and Benjamin 2003). However, long-term use of these drugs targeting the tumor vasculature may lead to hypoxia in the TME which re-activates VEGF expression and triggers angiogenesis resulting in more aggressive tumor growth (Awasthi et al., 2015). Given the heterogeneity in the regulation of angiogenesis, multidrug treatments targeting different angiogenic factors have been suggested as a more prudent approach than monotherapy to counter angiogenesis-mediated tumor growth (Rhim et al., 2014).

## 7 Conclusion

In this review, we described the current knowledge regarding the PDAC stromal components with special reference to collagens and mentioned their multifaceted role in disease development even from early stages. We emphasized the importance of these early changes in the ECM and their potential for being early detection markers in the blood. We also mentioned the desmoplastic reaction that takes place in the primary tumor and the metastatic site and explained its importance in tumor characteristics. We then discussed the role of acquired mechanical traits of the ECM and mechanoreciprocal interactions taking place within the TME and their function in the disease progression of PDAC. Furthermore, we pointed out the therapeutic opportunities that arise by studying the physical interactions within the TME. Moreover, we discussed the advancements of the models to study tumor-stromal interactions and their abilities to capture the physical characteristics of the TME.

Taken together, biochemical and physical interactions in the TME, affect the course of the disease and have been under the spotlight of research in recent years. Studies of these physical interactions can lead to the development of new tools for early detection and late-stage therapy options in the near future. Indeed, measuring circulating collagen fragments is an effective means by which to monitor disease progression (Willumsen et al., 2019), it also indicates that the composition and regulation of the ECM are dynamic and alter throughout disease progression. It would be interesting and potentially extremely useful to further study whether the composition of these secreted collagen fragments demonstrated any specificity to early PDAC or indeed for any specific fibrotic disease.

With respect to therapeutic avenues, it is clear that the desmoplastic reaction is a key hurdle to overcome for therapeutic perfusion into the tumor. An idea to explore in this content might be to target the cross-linking of the collagens in the tumor stroma in order to reduce the stiffness and improve drug delivery. Another idea might be to block the HAS enzymes in order to reduce the production of hyaluronan, however, its depletion has shown controversial results in the past (Hingorani et al., 2018) (Van Cutsem et al., 2020). Similarly, though it is encouraging to see that there are approaches that alleviate the severity of desmoplasia by targeting CAFs such as in cholangiocarcinoma (Nicolásnicolás-Boluda et al., 2020), it seems that PDAC is a case where ablating α-SMA expressing-CAFs results in worse outcomes (Rhim et al., 2014) (Özdemir et al., 2014) despite a corresponding reduction in fibrosis and associated desmoplasia. There ultimately remains a need to better understand the role of the ECM in the PDAC TME in order to explain this counter-intuitive finding and ultimately develop effective therapeutics for PDAC.

The effects of desmoplasia at the tissue scale are reasonably characterized (Figure 2), however how these biomechanics influence PDAC at the cellular level remains relatively poorly characterized. That said, the recent development of 3D PDAC cell and organoid culture models in tunable and dynamic hydrogel systems provide the perfect opportunity to decouple the biochemical and biomechanical properties of the ECM and identify any specific pathways ripe for therapeutic intervention.

Overall, the stroma is clearly an important component from potentially even prior to tumor initiation, through tumor development and eventual metastasis. Looking forward, with the development of investigative and analytical tools and approaches, the ECM and the TME provide much potential for the discovery of both diagnostic and therapeutic approaches. Further, it should be stressed that a focus on ablation of aspects of the PDAC TME has so far not been entirely fruitful, however there has been identification of CAF subtypes that have a tumor restraining phenotype. Perhaps in the future a focus on potentiation of tumor suppressive functions, rather than ablation, of the stroma will be an avenue to tame and treat the disease.
